# Transmission history of SARS-CoV-2 in humans and white-tailed deer

**DOI:** 10.1038/s41598-022-16071-z

**Published:** 2022-07-15

**Authors:** Katriina Willgert, Xavier Didelot, Meera Surendran-Nair, Suresh V. Kuchipudi, Rachel M. Ruden, Michele Yon, Ruth H. Nissly, Kurt J. Vandegrift, Rahul K. Nelli, Lingling Li, Bhushan M. Jayarao, Nicole Levine, Randall J. Olsen, James J. Davis, James M. Musser, Peter J. Hudson, Vivek Kapur, Andrew J. K. Conlan

**Affiliations:** 1 Disease Dynamics Unit (DDU), Department of Veterinary Medicine, University of Cambridge, Cambridge, UK; 2 School of Life Sciences and Department of Statistics, University of Warwick, Coventry, UK; 3 Animal Diagnostic Laboratory, Department of Veterinary and Biomedical Sciences, The Pennsylvania State University, University Park, PA 16802 USA; 4 Huck Institutes of Life Sciences, The Pennsylvania State University, University Park, PA 16802 USA; 5 Wildlife Bureau, Iowa Department of Natural Resources, Des Moines, IA USA; 6 Department of Veterinary Diagnostic and Production Animal Medicine, College of Veterinary Medicine, Iowa State University, Ames, IA USA; 7 The Center for Infectious Disease Dynamics, Department of Biology and Huck Institutes of the Life Sciences, The Pennsylvania State University, University Park, PA 16802 USA; 8 Department of Animal Science, The Pennsylvania State University, University Park, PA 16802 USA; 9 Laboratory of Molecular and Translational Human Infectious Disease Research, Center for Infectious Diseases, Department of Pathology and Genomic Medicine, Houston Methodist Research Institute, Houston Methodist Hospital, Houston, TX 77030 USA; 10 Department of Pathology and Laboratory Medicine, Weill Cornell Medical College, New York, NY 10021 USA; 11 Department of Microbiology and Immunology, Weill Cornell Medical College, New York, NY 10021 USA; 12 University of Chicago Consortium for Advanced Science and Engineering, University of Chicago, Chicago, USA; 13 Division of Data Science and Learning, Argonne National Laboratory, Lemont, IL 60439 USA

**Keywords:** Infectious diseases, Genomics, SARS-CoV-2, Viral epidemiology

## Abstract

The emergence of a novel pathogen in a susceptible population can cause rapid spread of infection. High prevalence of SARS-CoV-2 infection in white-tailed deer (*Odocoileus virginianus*) has been reported in multiple locations, likely resulting from several human-to-deer spillover events followed by deer-to-deer transmission. Knowledge of the risk and direction of SARS-CoV-2 transmission between humans and potential reservoir hosts is essential for effective disease control and prioritisation of interventions. Using genomic data, we reconstruct the transmission history of SARS-CoV-2 in humans and deer, estimate the case finding rate and attempt to infer relative rates of transmission between species. We found no evidence of direct or indirect transmission from deer to human. However, with an estimated case finding rate of only 4.2%, spillback to humans cannot be ruled out. The extensive transmission of SARS-CoV-2 within deer populations and the large number of unsampled cases highlights the need for active surveillance at the human–animal interface.

## Introduction

The emergence of a novel pathogen in a susceptible population with no previous exposure to the disease can result in rapid spread of infection with considerable associated morbidity and mortality^1,2^. Severe acute respiratory syndrome coronavirus 2 (SARS-CoV-2) was first observed in December 2019 in human patients with respiratory disease^3^. SARS-CoV-2 has subsequently spread globally and, by March 2022, caused over 6 million reported deaths in humans^4^.

SARS-CoV-2 is believed to have emerged from a zoonotic origin but the original animal reservoir remains unknown^5–9^. The high incidence of infection in humans during the ongoing pandemic poses a risk of anthropogenic transmission of SARS-CoV-2 to domestic animals and wildlife. Animals infected with SARS-CoV-2 have the potential to become reservoirs for infection and contribute to viral evolution and adaptation^10–12^. Since its emergence, natural SARS-CoV-2 infection has been documented in multiple animal species, including domestic animals, such as dogs (*Canis lupus familiaris*), cats (*Felis catus*), ferrets (*Mustela putorius furo*), and farmed American mink (*Neovison vison*), as well as captive wildlife in zoo settings^13–18^. Reported clinical presentation in infected animals has varied widely, ranging from subclinical infection to moderate respiratory or gastrointestinal signs in cats and dogs^13–15,19^, and increased respiratory disease and mortality in American mink populations^16^. The majority of SARS-CoV-2 infections reported in animals have been isolated spillover events to animals living in close contact with people with self-limiting transmission^14,15^ or onward transmission limited to animals housed together^13,15^. On the contrary, in farmed American mink, extensive transmission of SARS-CoV-2 occurred within farms as well as likely zoonotic spillover to farm workers^15,16^.

White-tailed deer (*Odocoileus virginianus*) are a free-living wild animal species present across the Americas and often live in close proximity to humans^20^, meaning deer could be exposed to SARS-CoV-2 in people through direct or indirect contact and become a source of spillback to humans. In addition, wildlife infected with SARS-CoV-2 could function as reservoir hosts for infection of other wildlife species or domestic animals^21^. White-tailed fawns experimentally infected with SARS-CoV-2 developed a subclinical viral infection and transmitted the infection to fawns in close proximity. Apart from a transient increase in body temperature in some of the fawns, no clinical signs were observed in the experimentally infected deer or those in indirect contact^22,23^. Viral RNA was shed in nasal secretions and faeces, with viral RNA persisting in nasal secretions in the majority of fawns when the study was discontinued at day 21 or day 22 post-inoculation or contact. However, no infectious SARS-CoV-2 was observed after day 6 or 7 post-infection^22,23^.

Recently, widespread SARS-CoV-2 infection and transmission was reported in white-tailed deer in Iowa, United States, with 33.2% testing positive overall on real-time reverse transcription-polymerase chain reaction (RT-PCR) from retropharyngeal lymph node samples^24^. From experimental challenge studies, it is known white-tailed deer can test positive for SARS-CoV-2 from retropharyngeal lymph node samples within two days of exposure to at least 3 weeks post challenge^22,23^. Thus, in contrast to samples obtained from nasal swabs in humans with clinical symptoms, positivity in deer could be an indicator of concurrent infection or previous exposure to SARS-CoV-2 virus rather than active infection at the time of testing.

Geospatial and phylogenetic clustering of deer SARS-CoV-2 cases suggest multiple human-to-deer spillover events followed by deer-to-deer transmission^24^. In addition, 35.8% of white-tailed deer tested in Ohio, United States, in January-March 2021 were infected with SARS-CoV-2^11^ and the U. S. Department of Agriculture (USDA) has confirmed SARS-CoV-2 in deer in multiple states^25^. SARS-CoV-2 infection has also been confirmed in deer in Canada, with possible spillback to humans^26,27^. Exposure to SARS-CoV-2 appears to be high in white-tailed deer populations, with 40% of white-tailed deer from various regions in the United States having antibodies for SARS-CoV-2^10^.

Knowledge of the direction and relative risk of disease transmission between competent hosts is essential to inform appropriate risk management strategies for SARS-CoV-2. Dated phylogenies from whole-genome sequence data can, in principle, be used to infer transmission networks both within and between species. Here, we use a transmission inference method^28^ to analyse a recently published dataset of SARS-CoV-2 genome sequences from deer^24^ and publicly available SARS-CoV-2 genomes from humans in Iowa to reconstruct the transmission dynamics of multiple spillover events and assess the relative risk of within and between species transmission. Given the uncertainty in the sampling time relative to infection for deer isolates and lack of quantitative data on the generation time for natural infection, we carried out a sensitivity analysis to explore the extent to which these quantities are identifiable from the genomic data alone.

## Methods

### Sample selection, processing and genome sequencing

The SARS-CoV-2 genomic data used in this study were from a recently published dataset described in Kuchipudi et al.^24^. Briefly, samples of medial retropharyngeal lymph nodes from white-tailed deer in Iowa, United States, were collected during routine surveillance for chronic wasting disease and tested for presence of SARS-CoV-2 RNA using RT-PCR. Whole-genome sequencing was performed with a NovaSeq6000 instrument (Illumina) on samples testing positive for SARS-CoV-2 viral RNA. A single-nucleotide polymorphism (SNP) based phylogenetic tree was constructed from the deer isolates and human SARS-CoV-2 sequences from Iowa during the same time period publicly available from Global Initiative on Sharing Avian Influenza Data (GISAID) as described by Kuchipudi et al.^24^. The largest cluster was selected for further analysis, consisting of 52 deer sequences collected between 28 September 2020 and 9 January 2021 and 141 human sequences from 22 July 2020 to 28 February 2021.

### Phylogenetic reconstruction

Using IQ-Tree 2.1.3 model selection method ModelFinder^29^, the generalised time reversible (GTR) substitution model was identified as the best-fit substitution model for the data based on the lowest Bayesian information criterion (BIC) score. A maximum likelihood tree of the selected cluster was generated in IQ-Tree 2.1.3. The presence of a temporal signal was assessed for the deer and human samples by comparing the root-to-tip distances against the sampling dates. The slope, correlation coefficient and R^2^ value were calculated (supplementary material, Fig. S1A) using R package phytools^30^, showing a significant but weak positive correlation (supplementary material, Fig. S1A).

We used BEAST 1.10.4 to construct dated phylogenetic trees, using sampling dates accurate to the day. The GTR nucleotide substitution model was adopted with a gamma-distributed site heterogeneity. We ran three Markov chain Monte Carlo (MCMC) runs for 100,000,000 iterations each, sampling every 10,000th iteration. To identify the most appropriate molecular clock and coalescent tree prior model for our data, different combinations of molecular clock and population priors were assessed, considering a strict or relaxed molecular clock and a constant, exponential or skyline population prior (supplementary material, Table S1). We assessed the performance of the models through posterior marginal likelihood estimates using path sampling (PS) and stepping-stone sampling (SSS), which measure the fit of the model to the data and have been shown to produce more consistent and reliable model selection than other marginal likelihood estimates such as harmonic mean estimator (HME) and Akaike’s information criterion (AIC)^31–33^. The model with the greatest marginal likelihood was chosen^31^, namely a strict molecular clock with a skyline coalescent population (supplementary material, Table S1). The three selected MCMC runs were combined in LogCombiner 1.10.4, with 10% of the chains removed as burn-in. Convergence of MCMC chains was assessed in Tracer 1.7.2, with a posterior effective sample size (ESS) of greater than 200 for each parameter. A maximum clade credibility (MCC) tree was then constructed in TreeAnnotator 1.10.4, using common ancestor node heights to enforce that all branches have positive lengths.

Due to the weak temporal signal observed in the root-to-rip distance assessment, a date randomization test (DRT) was performed to confirm the temporal signal using R package TipDatingBeast^34^. In the DRT, sampling dates are randomly reassigned to molecular data to create substitution rate estimates with no temporal signal^35^. A total of 20 datasets with randomly assigned tip dates were generated and BEAST was run on the datasets using the molecular clock and population model selected above. The 95% credible interval of the substitution rate estimates of the observed data was then compared to the 95% credible interval of the data with randomized dates, where no overlap suggests the temporal signal in the observed data was not obtained by chance^35^. The date randomization test showed no overlap between the 95% highest posterior density (HDP) credible interval of the real and date-randomized datasets, supporting presence of a temporal signal in the observed data (supplementary material, Fig. S1B).

### Inference of transmission

The transmission history was reconstructed from this dated tree using the R package TransPhylo 1.4.8^28^. As well as inferring who infected whom along the transmission chain, TransPhylo estimates the number of unsampled cases, providing an indication of how completely an outbreak has been sampled^28^ and allowing identification of subpopulations with unidentified cases that could be targeted for surveillance^36^. In addition to the dated phylogeny, TransPhylo requires information on the generation and sampling times specified as prior distributions. The generation time is defined as the interval between infection and onward transmission while the sampling time is the time from infection to sampling if the case is sampled. Based on estimates by Ganyani et al.^37^, we used a gamma prior with a mean of 5.2 days and standard deviation of 1.72 days for both the generation time and the sampling time distributions. TransPhylo was run over 500,000 iterations, sampling every 5th iteration and discarding 25% of the MCMC chain as burn-in. MCMC mixing and convergence were assessed by visual examination of the MCMC traces and ESS values greater than 100 for the within-host coalescent parameter (the product of the within-host population size and replication time, ${N}_{e}g$), reproduction number (*R*) and sampling probability (*π*) using the R package Coda^38^.

### Generation time and sampling time by species

The generation time can be inferred from the time interval between two cases becoming infected in the transmission chain. However, since the species of cases is only known for sampled deer and humans, the generation time was estimated for the infectors of sampled cases, where we assumed each infector of a sampled case was the same species as the sampled case. The posterior generation time was then calculated for deer and human separately by subtracting the inferred infection time of the infector of sampled hosts from the estimated time of infection of sampled hosts. The time from infection to sampling of an individual was estimated by species from the time of inferred infection of sampled cases to the time of sampling of the case.

### Sensitivity analysis of prior assumptions on generation and sampling time

Although experimental challenge studies suggest that the generation time in deer is broadly comparable to humans^22,23^, the time period between infection and sampling could be prolonged in deer if SARS-CoV-2 persists in retropharyngeal lymph nodes, resulting in very different sampling time distributions. To explore whether a difference in the generation and sampling time distributions between hosts can be inferred from the genomic data alone, we carried out a sensitivity analysis to the prior distributions (supplementary material, Table S2). This also allowed us to assess the effect of prior choices on the results, and therefore the robustness of inferred transmission networks to our prior assumptions.

## Results

**Figure 1:**
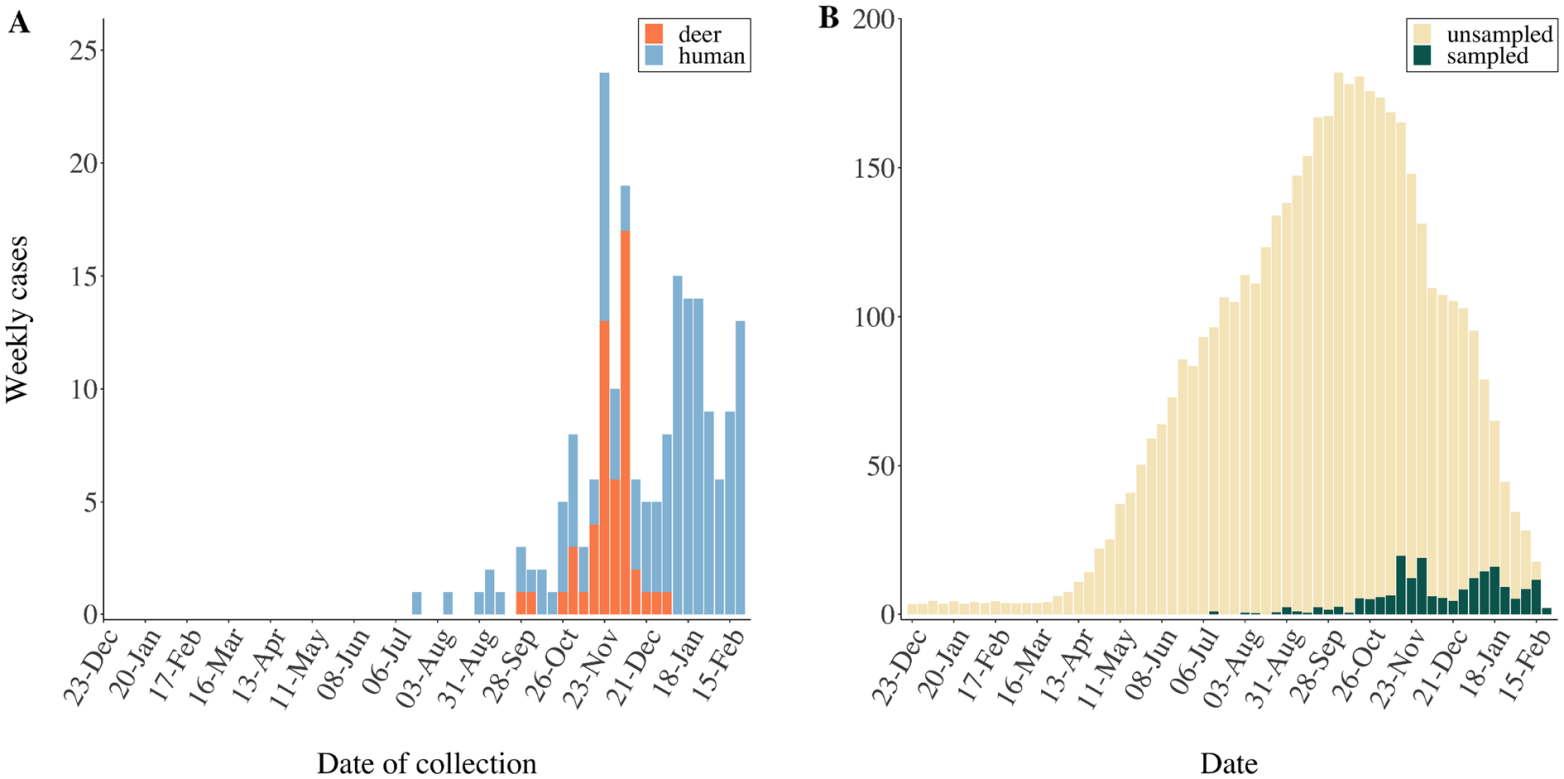
(**A**) Number of deer (orange) and human (blue) SARS-CoV-2 cases sampled per week between 22 July 2020 and 28 February 2021. (**B**) Mean inferred number of unsampled (beige) and sampled (green) cases in the transmission tree over time.

The cluster of cases selected for transmission analysis consisted of 52 deer SARS-CoV-2 samples collected between 28 September 2020–9 January 2021 and 141 human samples from 22 July 2020–28 February 2021 (Fig. 1A). From this set of 193 whole-genome SNP sequences, BEAST estimated the time of the most recent common ancestor for human sequences to be 209 days before the first sampled case, corresponding to 26 December 2019 (95% HDP credible interval = 92–333 days before first sampled isolate, ESS = 1503). The most recent common ancestor for deer sequences was estimated to have existed 113 days before the first sampled case, corresponding to 31 March 2020 (95% HDP interval = 41–196 days, ESS = 1079), suggesting that transmission within deer populations could have been sustained before the high levels of infection observed during the hunting season (September to January) when the majority of samples were submitted. The estimated SARS-CoV-2 effective population size showed a steep increase in early October 2020 when considering both deer and human cases together compared to human samples alone which increased more gradually (supplementary material, Fig. S3). Nevertheless, cases ancestral to the SARS-CoV-2 sequences from deer could include both human and deer infections.

**Figure 2:**
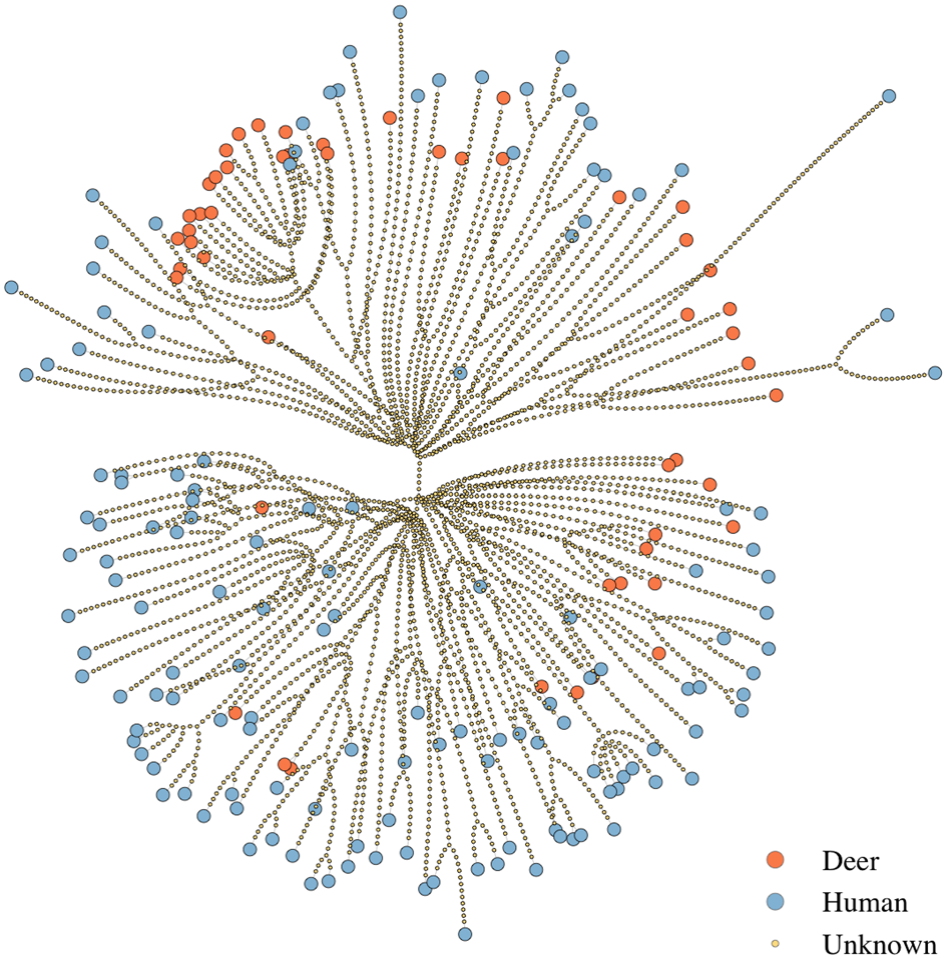
Medoid inferred transmission network for SARS-CoV-2 cases in white-tailed deer (orange) and humans (blue) in Iowa between July 2020 and February 2021. Nodes represent cases and edges represent transmission events. Unsampled cases are shown as smaller nodes and correspond to inferred transmission intermediates.

The generated MCC phylogenetic tree (supplementary material, Fig. S2A) shows several clusters and single cases of SARS-CoV-2 from deer interspersed among human isolates, consistent with multiple spillover events from human to deer as previously described in Kuchipudi et al.^24^. This is also evident from the medoid transmission tree (Fig. 2; supplementary material, Fig. S2B), which corresponds to the inferred transmission tree that is most similar to all posterior sampled transmission trees^39^. The mean terminal branch length was slightly higher for sampled human cases (76 days, range: 7–275) than deer (61 days, range: 8–156).

The reproduction number (*R*), corresponding to the number of secondary cases resulting from an infected individual^39^, was estimated to be 1.05 (supplementary material, Table S2). This inferred *R* slightly above one suggests that the outbreak was growing exponentially at a slow rate from the moment it started in December 2019 until the end of the sampling period in February 2021. Nevertheless, the estimated value of the reproduction number is an average over this time period and may hide subphases of growth and decline.

**Figure 3:**
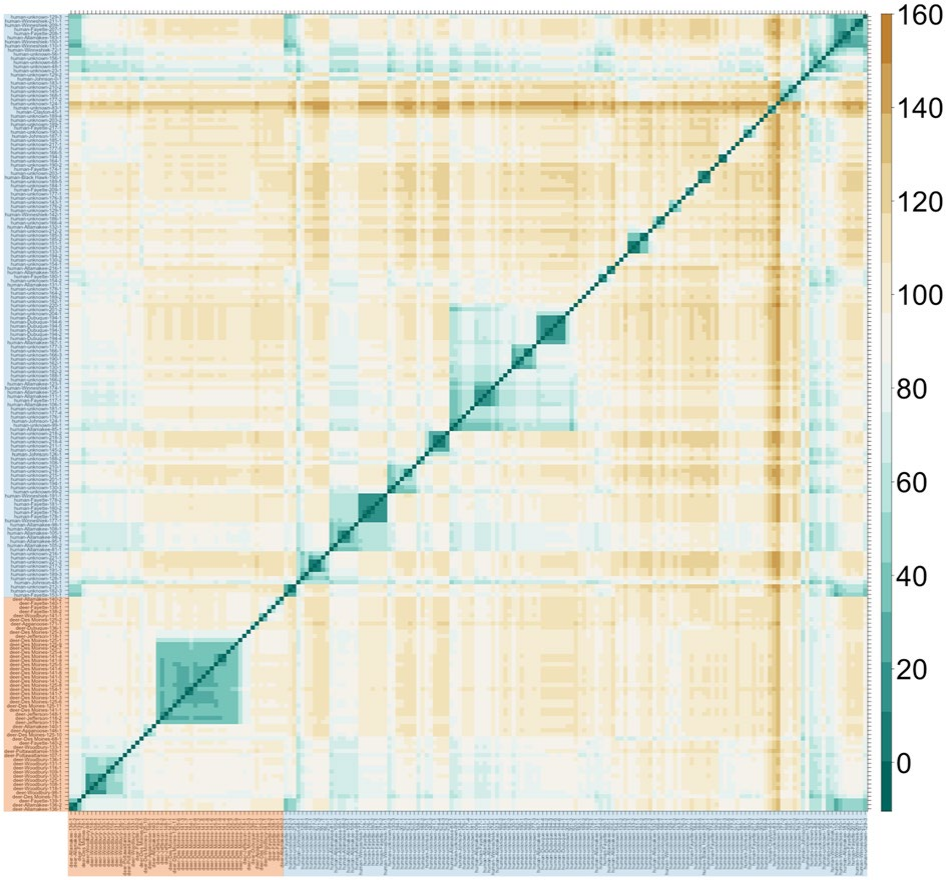
Inferred number of intermediate cases in the transmission chain between each pair of sampled cases. Cases are shown on the left vertical and horizontal axes, where deer cases are highlighted in orange and human cases in blue. The colour scale of the number of intermediate cases is indicated on the right vertical axis.

The estimated number of unsampled cases in the transmission tree was high (Figs. 1B, 2), with a median of 4394 (range: 4337–4460) inferred unsampled cases, corresponding to a case finding rate of 4.2% (range: 4.1–4.3%). The true case finding rate will be even lower, since unsampled cases that do not result in at least one sampled case are not represented in the transmission tree^39^. The estimated mean posterior sampling probability (*π*) was 0.1% (supplementary material, Table S2). Between each pair of sampled cases, the number of intermediates in the transmission chain ranged between two and 150 individuals, with a median of 97 intermediates, also indicative of a low case finding. The inferred number of intermediate cases in the transmission chain between each pair of sampled cases is illustrated in Fig. 3. This number represents the average number of cases on the path between two sampled cases on all reconstructed transmission trees, including, for example, the medoid tree shown in Fig. 2. The high number of unsampled cases was further highlighted by the probabilities of direct transmission between sampled cases being low, with no transmission pairs having a higher than 50% probability of direct transmission. There was no supported transmission pair identified between species.

**Figure 4:**
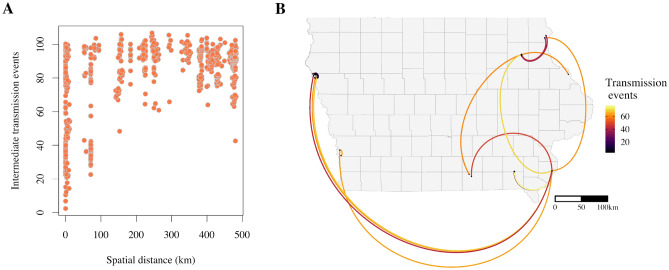
(**A**) Geographic distance and mean inferred number of transmission events between each pair of sampled deer. (**B**) Map of Iowa, United States, showing the geographical location of SARS-CoV-2 deer samples and links to the nearest deer in number of transmission events.

A larger number of intermediate transmission events was observed between deer separated by a greater spatial distance (Fig. 4A). Nevertheless, some of the deer clusters contained samples from multiple, geographically divergent counties in Iowa, with the most notable one containing SARS-CoV-2 deer samples from Fayette (northeast Iowa), Allamakee (northeast Iowa), Woodbury (west Iowa) and Des Moines (southeast Iowa) Counties (supplementary material, Fig. S2), corresponding to three separate regions of the state. All but one county with SARS-CoV-2 positive deer from the analysed sequence cluster had at least one case with transmission links to Des Moines County when the nearest sampled deer in the transmission pathway was assessed (Fig. 4B).

**Figure 5:**
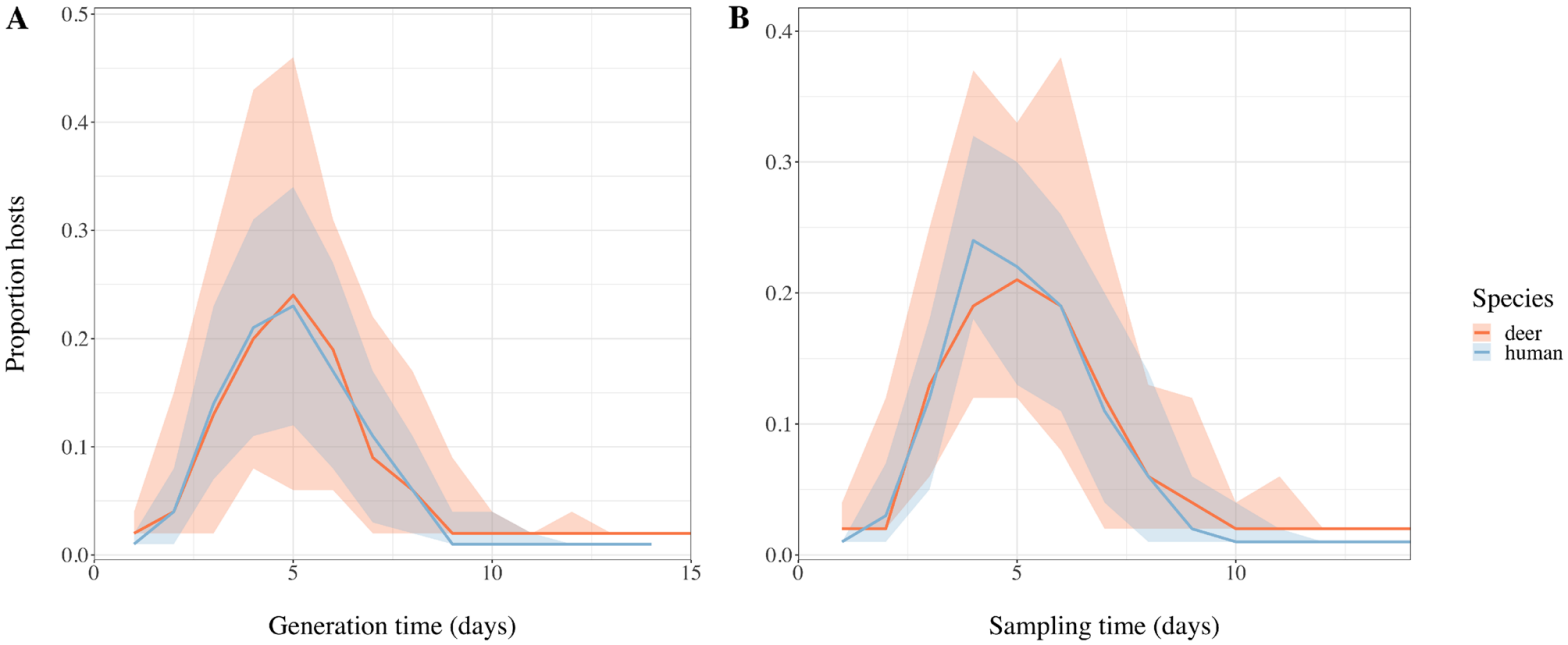
(**A**) Posterior median generation time of SARS-CoV-2 and (**B**) posterior median time between becoming infected with SARS-CoV-2 and being sampled with associated range (shaded area) for sampled deer (orange) and humans (blue).

The median estimated generation time, i.e. the time interval between infection of an individual and onward transmission, was five days for both deer and humans, ranging from one to 15 days for deer and one to 14 days for humans (Fig. 5A). There did not appear to be a substantial difference in the time elapsing between becoming infected and being sampled for sampled deer and humans (Fig. 5B). The time from becoming infected to being sampled ranged between one to 14 days for deer and humans, with a median sampling time of five days for both species. We performed a sensitivity analysis on the prior assumptions of the generation time and sampling time to explore whether a prolonged generation or sampling time may be occurring in deer. Extending the priors did not result in divergent estimates for the generation time or sampling time between deer and humans. Nevertheless, the inferred generation and sampling time appeared highly sensitive to the specified prior distributions (supplementary material, Fig. S4).

## Discussion

In this study, whole-genome sequencing data were combined with epidemiological parameters to further characterise the transmission of SARS-CoV-2 between human and deer populations. Our results are consistent with multiple spillover events from human to deer with likely onward transmission within deer populations. As reported in Kuchipudi et al.^24^, we found no evidence of deer-to-human transmission within our sample set. However, due to the high number of unsampled cases, deer-to-human transmission cannot be ruled out. To infer transmission events at the human–animal interface, additional surveillance in deer would have to be complemented by further identification and sequencing of SARS-CoV-2 cases in humans. Individuals more likely to come into contact with deer populations, such as hunters and hikers in deer habitats, could be targeted for surveillance to identify any high-risk populations for spillover events. All deer cases were at the end of the inferred transmission chain (Fig. 2), so further assessment into the number of secondary cases of deer and if variation occurs based on age or sex could not be made. However, once more data becomes available, risk factors for onward transmission within deer populations should be revisited.

The deer samples assessed were recovered from hunted deer, roadkill or deer targeted due to poor health condition. In the late 2000s, there were an estimated 330,000–475,000 deer in Iowa^40^ and 109,544 deer were reported harvested in the 2020–2021 hunting season^41^, so the 283 deer evaluated in Kuchipudi et al.^24^ only makes up a small proportion of the population and a high number of unsampled SARS-CoV-2 deer cases was expected. Nevertheless, for certain groups of deer, there appeared to be a lower number of intermediate cases than for humans (Fig. 3), suggesting a higher proportion of deer cases of SARS-CoV-2 might be sampled and sequenced than human cases. The low sampling proportion in humans may be a combination of asymptomatic infection, symptomatic individuals not getting tested, positive cases not being sequenced, or human SARS-CoV-2 sequences not being made publicly available.

It is currently unknown for how long viral RNA can be found in retropharyngeal lymph nodes of deer following exposure to infection. In experimentally infected deer, SARS-CoV-2 RNA was found in tissues collected on day 21 or 22, including retropharyngeal lymph nodes, while infectious SARS-CoV-2 assessed by virus isolation in nasal secretions was only detected between day one and six or seven post-inoculation or contact^22,23^. Consequently, deer testing positive for SARS-CoV-2 from retropharyngeal lymph node samples could have experienced previous infection with virus remaining in the lymph nodes for a longer time period rather than having an actively shedding viral infection. The time from infection to sampling would, therefore, be expected to vary between humans and deer as the timing of sampling in deer will not coincide with onset of symptoms or testing as part of contact tracing. If viral RNA can persist within retropharyngeal lymph nodes after deer have recovered from infection, we would expect to observe a longer sampling time in some deer. Although the predicted range for sampling time was slightly wider in deer, the inferred time from infection to sampling in deer was not extended and the sampling time followed a similar distribution for deer and humans (Fig. 5; supplementary material, Fig. S4). Similarly, we found no evidence for a diverging generation time for deer and humans (supplementary material, Fig. S4). The priors used for the generation time were based on estimates in humans^37^ but there are currently no comparable estimates for naturally infected deer. However, prolonging the generation and sampling time priors suggested the estimated posterior generation time and sampling time were sensitive to the specified priors (supplementary material, Table S2; supplementary material, Fig. S4) highlighting the necessity of independent data to estimate both the serial interval in deer and relationship to lymph node positivity.

Since there was limited evidence for direct transmission between sampled cases and the species of unsampled cases is unknown, we assumed the infector of sampled cases was the same species as the sampled individual to estimate the generation time by species. This was further supported by multiple single species clusters (Fig. 2; supplementary material, Fig. S2), suggesting within-species spread. Nevertheless, a higher sampling density would be needed to identify direct transmission events between cases and to confirm the species of infectors, reiterating the importance of developing a robust SARS-CoV-2 surveillance program in white-tailed deer with regular sampling.

Increased contact rates between animals living in groups could facilitate rapid pathogen spread within a susceptible population over a short period of time. Deer herd structure and contact patterns vary over the year and are highly seasonal^42^. Female white-tailed deer live in relatively stable social groups with multiple offspring for the majority of the year apart from during fawning, when they become solitary (reviewed in Schauber et al.^42^). Contact between female social groups mainly occurs during feeding, highlighting the potential role of feed availability on contact rates^43^. Male deer have larger home territories and more frequent social contacts than female deer, increasing the risk of exposure to pathogens (reviewed in Grear et al.^44^). While Kuchipudi et al.^24^ did not observe any significant difference in test positivity based on sex, Hale et al.^11^ found that male deer were more likely to test positive for SARS-CoV-2. There are high rates of movement in the fall during the breeding season, which varies by latitude and takes place between October and January in Iowa, with peak breeding season happening in November^41^. The breeding season coincides with the hunting season, which runs in sessions between September and January^45^, during which the majority of deer samples were collected for this study (Fig. 1A). Year-round surveillance for SARS-CoV-2 in white-tailed deer would reveal if the prevalence remains high through the year, exhibits a seasonal pattern or if it is purely spillover and a reflection of the human epidemic curve. The clustering of SARS-CoV-2 isolates in deer from distant geographical locations of Iowa (Fig. 4B; supplementary material, Fig. S2) warrants further assessment of potential transmission routes over longer distances and whether it is the outcome of human travel and engagement in activities with a high risk of spillover to deer in multiple locations, long distance movement of deer between counties, or through an alternative route of transmission.

The extensive transmission of SARS-CoV-2 in wild deer populations highlights the need for surveillance at the human–animal interface. Without active surveillance in susceptible wild animal populations, the virus could circulate and evolve undetected for extended periods of time. By inferring the transmission tree at the human–animal interface, one could identify potential at-risk populations or high-risk activities for transmission. However, to be able to follow the transmission pathway, cases need to be identified and sequenced. Already existing disease surveillance programmes in deer could be extended to include SARS-CoV-2 to monitor occurrence and mutations with potential to affect transmissibility, virulence or other adverse phenotypic changes. Furthermore, for more reliable inference of transmission events at the human–animal interface, surveillance in deer would have to be complemented by equivalent surveillance in the sympatric human population and independent data on the serial interval of natural infection in deer.

## Supplementary Information

Supplementary Information.

## Data Availability

Source code for models performed in R and generated figures is provided in GitHub repository https://github.com/kwillgert/SARS-CoV-2_deer.
