# Supplementary material for: Transmission history of SARS-CoV-2 in humans and white-tailed deer
Source: Sci Rep. 2022 Jul 15;12:12094. doi: 10.1038/s41598-022-16071-z (PMC9284484; doi:10.1038/s41598-022-16071-z)
Supplement: Supplementary file 1 — Supplementary Information. [file 41598_2022_16071_MOESM1_ESM.pdf]

Supplementary material for

Transmission history of SARS-CoV-2 in humans and white-tailed deer

Katriina Willgert[1*], Xavier Didelot[2], Meera Surendran-Nair[3, 4], Suresh V. Kuchipudi[3, 4], Rachel M. Ruden[5, 6], Michele Yon[3], Ruth H. Nissly[3, 4], Kurt J. Vandegrift[7], Rahul K. Nelli[6], Lingling Li[3], Bhushan M. Jayarao[3], Nicole Levine[4, 8], Randall J. Olsen[9, 10, 11], James J. Davis[12], James M. Musser[9, 10, 11], Peter J. Hudson[7], Vivek Kapur[4, 8], Andrew J. K. Conlan[1]

[1]*Disease Dynamics Unit (DDU), Department of Veterinary Medicine, University of Cambridge, United Kingdom;*
[2]*School of Life Sciences and Department of Statistics, University of Warwick, United Kingdom;*
[3]*Animal Diagnostic Laboratory, Department of Veterinary and Biomedical Sciences, The Pennsylvania State University, University Park, PA 16802, United States;*
[4]*Huck Institutes of Life Sciences, The Pennsylvania State University, University Park, PA 16802, United States;*
[5]*Wildlife Bureau, Iowa Department of Natural Resources, Des Moines, Iowa, USA,*
[6]*Department of Veterinary Diagnostic and Production Animal Medicine, College of Veterinary Medicine, Iowa State University, Ames, Iowa, USA,*
[7]*The Center for Infectious Disease Dynamics, Department of Biology and Huck Institutes of the Life Sciences, The Pennsylvania State University, University Park, PA 16802, United States;*
[8]*Department of Animal Science, The Pennsylvania State University, University Park, PA 16802, United States;*
[9]*Laboratory of Molecular and Translational Human Infectious Disease Research, Center for Infectious Diseases, Department of Pathology and Genomic Medicine, Houston Methodist Research Institute, Houston Methodist Hospital, Houston, TX 77030, United States;*
[10]*Department of Pathology and Laboratory Medicine, Weill Cornell Medical College, New York, NY 10021, United States;*
[11]*Department of Microbiology and Immunology, Weill Cornell Medical College, New York, NY 10021, United States;*
[12]*University of Chicago Consortium for Advanced Science and Engineering, University of Chicago and Division of Data Science and Learning, Argonne National Laboratory, Lemont, IL 60439, United States*

Supplementary tables

Table S1. Log-marginal likelihood estimates using path sampling (PS) and stepping-stone sampling (SSS) for model selection. The overall ranking of the models is shown in parenthesis.

**Clock**	**Coalescent prior**	**PS**	**SSS**
Strict	Constant	-6543.5 (5)	-6543.7 (5)
Strict	Exponential	-6500.2 (3)	-6499.9 (4)
Strict	Skyline	-6479.8 (1)	-6480.4 (1)
Uncorrelated*	Constant	-6545.5 (6)	-6545.9 (6)
Uncorrelated*	Exponential	-6500.2 (3)	-6498.9 (3)
Uncorrelated*	Skyline	-6486.3 (2)	-6486.9 (2)

* Uncorrelated = uncorrelated relaxed clock with log-normal distribution

Table S2. Priors considered for the generation time and sampling time (mean time) and standard deviation (SD) to assess whether there was a difference in the generation time (GT) and sampling time (ST) between humans and deer. For each scenario, the posterior mean and effective sample size (ESS) are reported for the within-host coalescent parameter $N_e g$, reproduction number (R) and sampling proportion (π), as well as the median number of unsampled cases, case finding (%), median generation time and median time from infection to sampling. GT = generation time, ST = sampling time.

Scenario		$N_e g$		R		π		Unsampled cases	Case finding (%)	Generation time		Sampling time	
Mean time (days)	SD	Mean	ESS	Mean	ESS	Mean	ESS			Deer	Human	Deer	Human
5.2	1.72	2.3	296	1.05	39824	0.001	22924	4394	4.2	5	5	5	5
5.2*2	1.72*2	9.2	659	1.12	20537	0.004	31680	2048	8.6	10	10	10	10
5.2*3	1.72*3	15.7	393	1.18	10586	0.009	26243	1255	13.3	15	16	15.5	16
GT: 5.2<br>ST: 20	1.72<br>20	2.7	174	1.06	21682	0.001	11579	4130	4.5	5	5	10	8

Supplementary figures

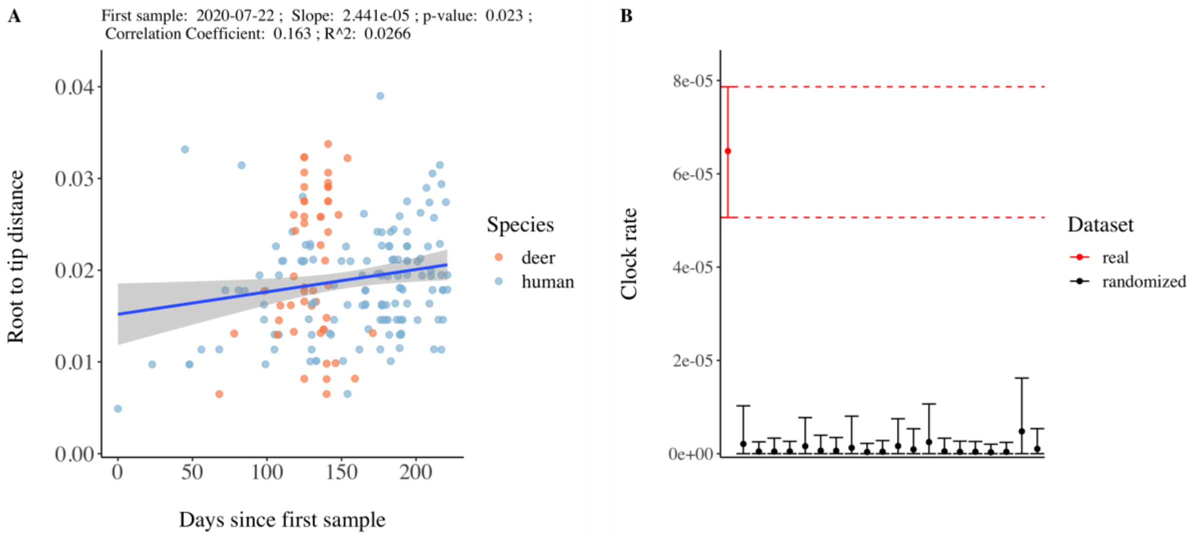

Figure S1. Temporal signal assessment. A) Plot of phylogenetic root-to-tip distance against the sampling date for deer (orange) and human (blue) samples and associated regression slope (blue line). The date of the first sample, slope, p-value, correlation coefficient and R^2 are indicated at the top. B) Date randomization test (DRT) comparing the substitution rates and 95% credible intervals estimated in BEAST for the original dataset (in red) and 20 datasets with permutated dates (in black). There was no overlap between the 95% credible interval of the real dataset (red bars, horizontal dashed line) and the date-randomized datasets (black bars).

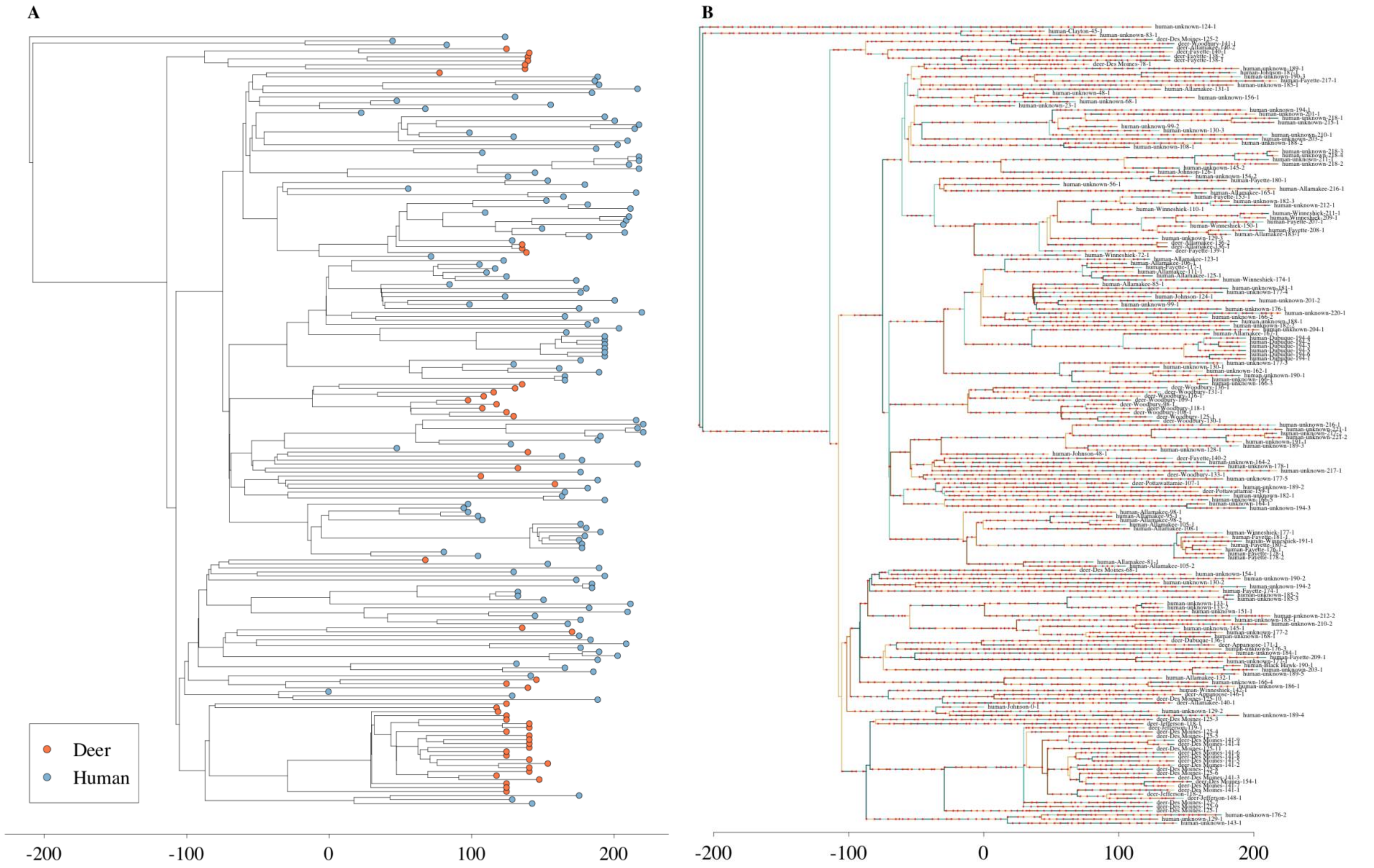
A
Deer
Human
-200
-100
0
100
200
B
-200
-100
0
100
200

Figure S2. A) Maximum clade credibility phylogenetic tree generated with a strict molecular clock and a skyline coalescent population from whole-genome SNP sequences of SARS-CoV-2 in white-tailed deer (orange) and humans (blue) in Iowa between July 2020 and February 2021. The phylogeny is dated (X-axis), where day 0 corresponds to the first collected sample on 22 July 2020. B) The most representative (medoid) transmission tree inferred in TransPhylo for SARS-CoV-2 cases in white-tailed deer and humans. Cases are indicated in different colours on the branches of the phylogenetic tree, where a colour change highlighted by a red asterisk corresponds to a transmission event. Sampled cases are at the end of the branches with host species indicated in the labels, whereas the species of unsampled cases along the branches is unknown.

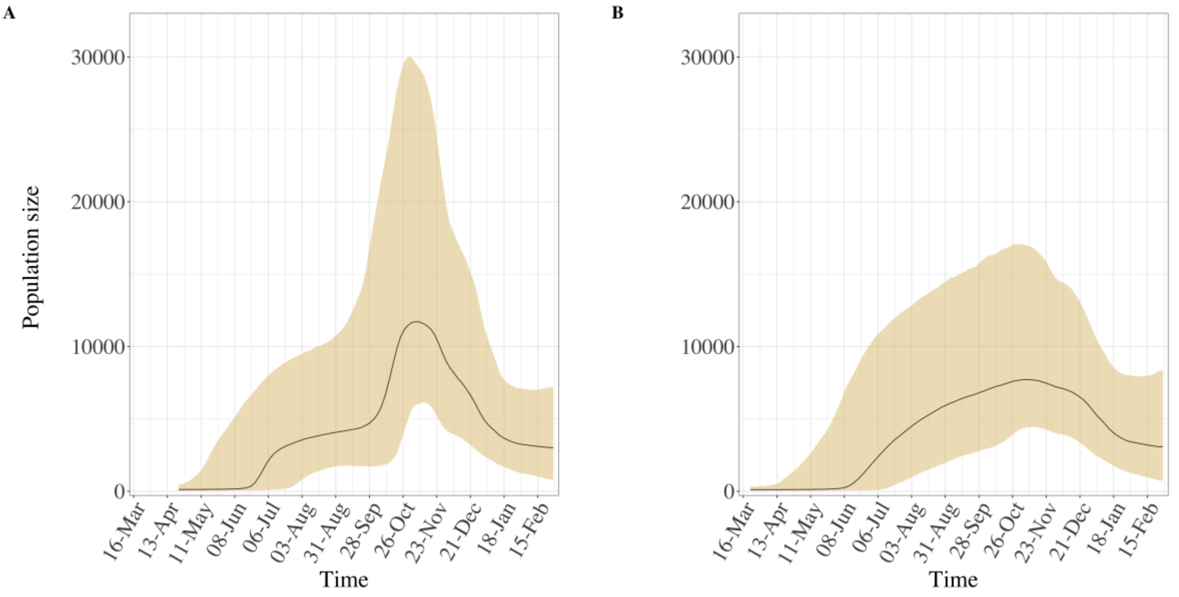

Figure S3. Coalescent Bayesian Skyline plot of the median estimated effective population size and 95% HPD interval (shaded area) of SARS-CoV-2 over time, where A) both human and deer SARS-CoV-2 sequences were considered to estimate the effective population size and B) human SARS-CoV-2 sequences only were used.

1) mean = 5.2 days, sd = 1.72 days

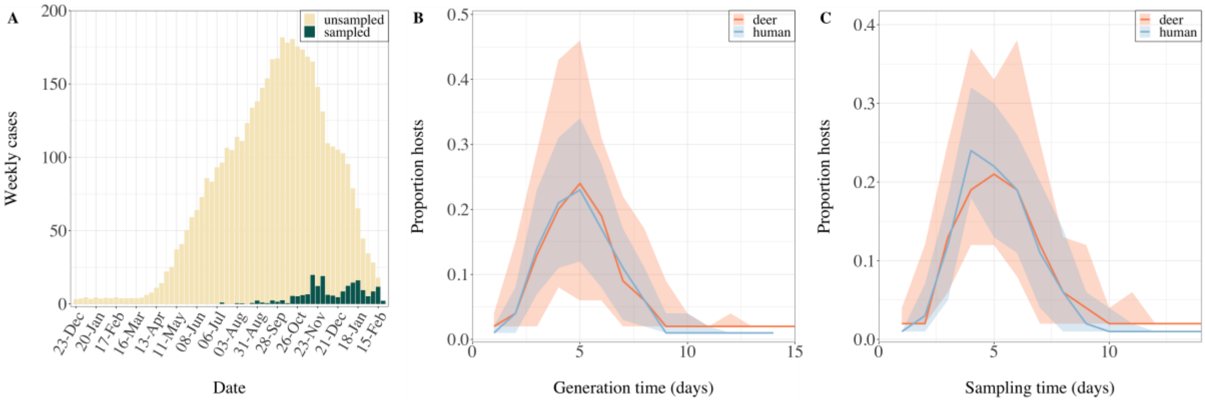

2) mean = 5.2*2 days, sd = 1.72 days

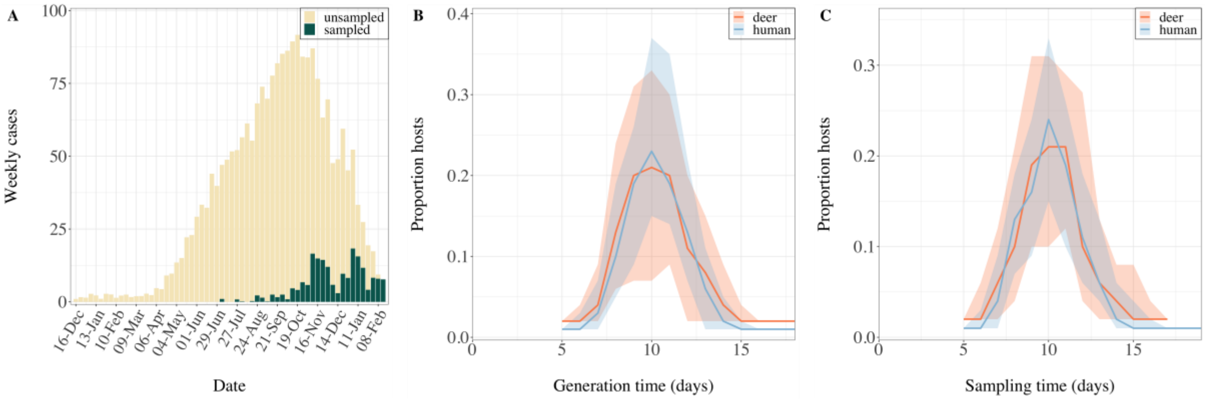

3) mean = 5.2*3 days, sd = 1.72 days

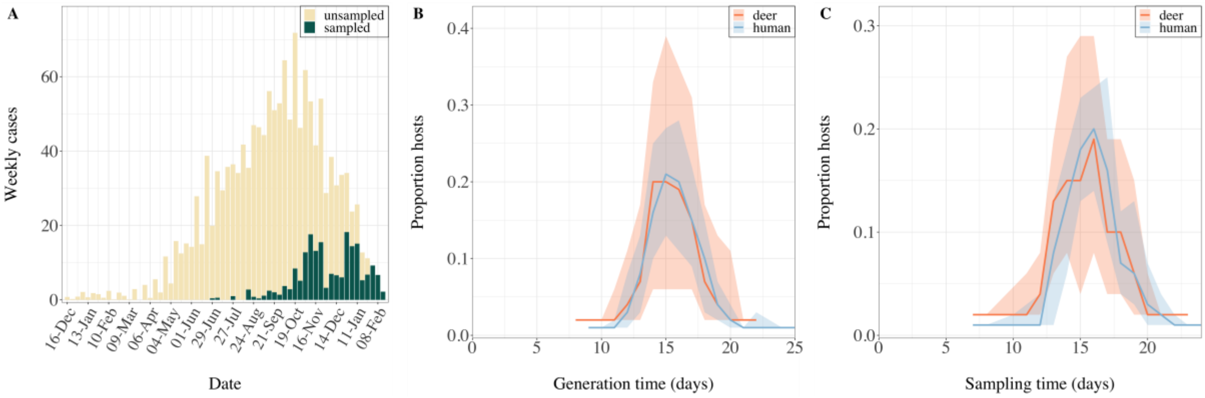

4) Generation time: mean = 5.2 days, sd = 1.72 days; Sampling Time: mean = 20 days, sd = 20 days

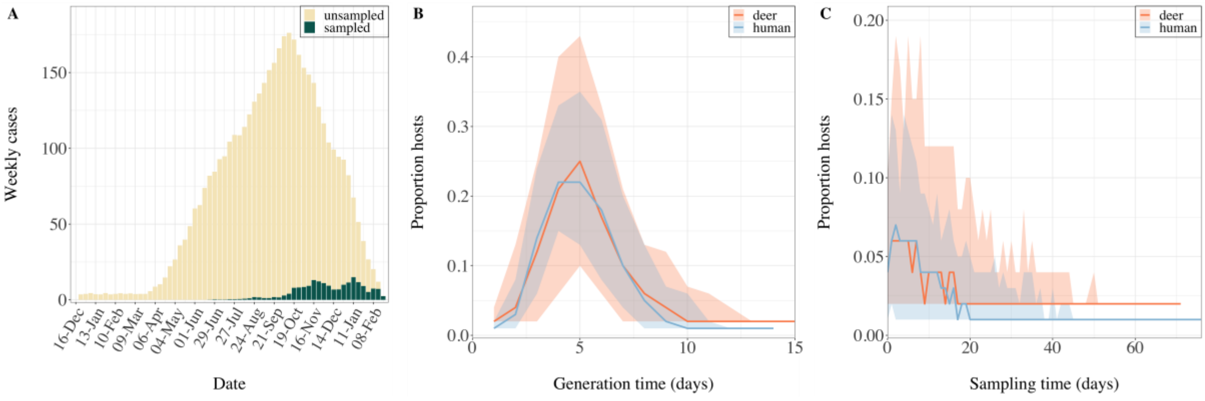

Figure S4. Sensitivity analysis of model assumptions for generation time and sampling time with four considered scenarios. For scenarios 1-3, the sampling time was set to the same distribution as the generation time. For scenario 4, an extended sampling time was assumed. For each scenario, mean inferred number of unsampled (beige) and sampled (green) cases over time (A), posterior median generation time of SARS-CoV-2 with associated range (shaded area) for sampled deer (orange) and humans (blue) (B) and posterior median time between becoming infected with SARS-CoV-2 and being sampled with associated range for sampled deer (orange) and humans (blue) (C) are reported.
